# Protein Glutathionylation in Cardiovascular Diseases

**DOI:** 10.3390/ijms141020845

**Published:** 2013-10-17

**Authors:** Anna Pastore, Fiorella Piemonte

**Affiliations:** 1 Laboratory of Biochemistry, Bambino Gesù Children’s Hospital, IRCCS, 00165 Rome, Italy; E-Mail: anna.pastore@opbg.net; 2 Unit of Neuromuscular and Neurodegenerative Diseases, Bambino Gesù Children’s Hospital, IRCCS, 00165 Rome, Italy

**Keywords:** glutathione, *S*-glutathionylation, myocardial, contraction, metabolism, hypertrophy, inflammation, cardiovascular diseases, atherosclerosis

## Abstract

The perturbation of thiol-disulfide homeostasis is an important consequence of many diseases, with redox signals implicated in several physio-pathological processes. A prevalent form of cysteine modification is the reversible formation of protein mixed disulfides with glutathione (*S*-glutathionylation). The abundance of glutathione in cells and the ready conversion of sulfenic acids to *S*-glutathione mixed disulfides supports the reversible protein *S*-glutathionylation as a common feature of redox signal transduction, able to regulate the activities of several redox sensitive proteins. In particular, protein *S*-glutathionylation is emerging as a critical signaling mechanism in cardiovascular diseases, because it regulates numerous physiological processes involved in cardiovascular homeostasis, including myocyte contraction, oxidative phosphorylation, protein synthesis, vasodilation, glycolytic metabolism and response to insulin. Thus, perturbations in protein glutathionylation status may contribute to the etiology of many cardiovascular diseases, such as myocardial infarction, cardiac hypertrophy and atherosclerosis. Various reports show the importance of oxidative cysteine modifications in modulating cardiovascular function. In this review, we illustrate tools and strategies to monitor protein *S*-glutathionylation and describe the proteins so far identified as glutathionylated in myocardial contraction, hypertrophy and inflammation.

## 1. Introduction

Oxidative stress represents an imbalance between ROS production and the cellular antioxidant defense system. In stress conditions, ROS levels increase and, because of their high reactivity, participate in a variety of chemical reactions. They are involved in cell damage, necrosis and apoptosis via oxidation of lipids, proteins and DNA and also provoke endothelial dysfunction, infiltration and activation of inflammatory cells [1]. ROS generation can be finely controlled and can constitute a physiologic signaling pathway, also mediating *S*-glutathionylation.

Protein glutathionylation is becoming increasingly recognized as playing a causative role in cardiovascular disorders (CVDs). Indeed, it regulates numerous physiological processes involved in cardiovascular homeostasis and/or perturbed in disease, including myocyte contraction, oxidative phosphorylation, protein synthesis, vasodilation, glycolytic metabolism and response to insulin. Therefore, perturbations in protein glutathionylation status may contribute to the etiology of cardiovascular diseases, such as myocardial infarction, cardiac hypertrophy and atherosclerosis. In this review, we discuss the glutathione-related modifications underlying redox signaling in CVDs focusing on myocardial contraction, metabolism, proliferation, hypertrophy and inflammation.

## 2. Protein-*S*-Glutathionylation Status in Cardiovascular Diseases

Protein *S*-glutathionylation is emerging as a critical signaling mechanism in cardiovascular diseases, because it regulates numerous physiological processes involved in cardiovascular homeostasis, including myocyte contraction, oxidative phosphorylation, protein synthesis, vasodilation, glycolytic metabolism and response to insulin [2–5]. Thus, perturbations in protein glutathionylation status may contribute to the etiology of cardiovascular diseases, such as myocardial infarction, cardiac hypertrophy and atherosclerosis.

### 2.1. Myocardial Infarction

Overall protein glutathionylation increases following ischemia-reperfusion (IR), with the majority of the glutathionylation events occurring early in the reperfusion period [6]. Glyceraldehydes-3-phosphate dehydrogenase (GAPDH) was identified as a prominent cardiac protein glutathionylated during IR with loss of enzyme function, suggesting that GAPDH glutathionylation is likely inhibitory *in vivo*. The effect of this inhibition may be: (a) a block of glycolysis characteristic of ischemic injury; (b) an interference with nuclear translocation, resulting in increased apoptosis or (c) a homeostatic answer to the excess of oxidants. Of note, GAPDH activity is restored at the end of the reperfusion, thus suggesting that glutathionylation may constitute a temporary protection of catalytic cysteines from irreversible oxidation. Chen and Ogut [7] further evidenced the glutathionylation of actin in a rat model of *in vivo* IR. Studies on isolated G-actin indicated that glutathionylation delayed its rate of polymerization and decreased the cooperativity of its binding to tropomyosin [8], suggesting that actin glutathionylation may contribute to the decline in cardiac contractility observed during ischemia. In contrast, mitochondrial complex II seems to be deglutathionylated during IR [9], indicating that a single oxidative stimulus can affect glutathionylation in different directions and highlighting also the potential critical role of Grx in myocardial infarction. Mouse models of embryonic *Grx1* knockout, as well as overexpression of Grx1 and Grx2 transgenes, were developed and subjected to *in vivo* and *ex vivo* IR [10–12]. Such experiments suggest a cardioprotective role for Grx isoforms, but additional studies are however needed. An attractive candidate in cardio-protection is mitochondrial complex I, in which glutathionylation of the 51- and 75-kD subunits is correlated with electron transport inhibition and increased production of superoxide [13]. Glutathionylation of complex I, with associated increases in superoxide production, would be expected to increase cytochrome c release and caspase activation, inducing survival signals and contributing to infarct size and cardiac dysfunction. The deglutathionylation of complex I by Grx2 could represent an upstream event responsible for modulating these effects in Grx2 transgenic animals.

### 2.2. Cardiac Hypertrophy

Multiple signaling pathways contribute to the development of pathological cardiac hypertrophy [14]. Among them, the Raf/MEK/ERK pathway can be stimulated either by G protein-coupled receptor ligands (e.g., angiotensin II, endothelin) or by mechanical stretch resulting in induction of protein synthesis. Pimentel *et al.* [15] showed that mechanical strain stimulating the Raf/MEK/ERK pathway was dependent upon glutathionylation of Ras in neonatal rat ventricular myocytes, a small GTPase implicated in myocyte growth signaling. The authors demonstrated that glutathionylated Ras was formed in response to a physiological stimulus (mechanical strain) and that glutathionylation induced the increase of Raf and GTP binding regulating the protein synthesis, important in cardiac hypertrophy. Thus, investigating the Ras glutathionylation status in animal models of cardiac hypertrophy may provide insight into the progression of the disease *in vivo*.

### 2.3. Atherosclerosis

The precise role of glutathionylation in the development and progression of atherosclerosis is unknown; however, conditions within atherosclerotic plaques (e.g., hypoxia, oxidative stress, oxidized LDL, and inflammation) have been shown to promote glutathionylation in other contexts [6,16,17], and Grx has been reported to associate with areas of oxidative stress within the vasculature [18].

Protein glutathionylation increases in human macrophages exposed to oxidized LDL (oxLDL), a major component of atherosclerotic plaques [17] and, together with glutathione (GSH) depletion, increased glutathionylated proteins seem to be implicated in oxLDL-induced macrophage death *in vitro* [19]. The role of specific glutathionylated proteins in macrophage cell death is not yet determined, nor is it known whether global protein glutathionylation increases in other cells types exposed to oxLDL. Patients with atherosclerosis of the extremities (*i.e*., atherosclerosis obliterans (ASO)) exhibit increased glutathionylation of serum proteins, and a positive correlation between disease progression and the level of protein glutathionylation was found [20]. The authors identified ApoB100, the major component of LDL, as a target for increased glutathionylation in ASO, but if the glutathionylated apoB100 represents a disease marker or contributes to the pathogenesis of ASO remains an open question.

Sarco(endo)plasmic reticulum Ca^2+^ ATPase (SERCA) glutathionylation represents a physiological, cGMP-independent mechanism of vessel relaxation, and it is disrupted during atherosclerosis [21]. Site-directed mutagenesis and mass spectroscopic analysis suggested that glutathionylation of Cys^674^, located in the cytosolic-facing hinge domain, was responsible for SERCA activation. The irreversible oxidation (*i.e*., sulfonic acid formation) of SERCA’s Cys^674^ during atherosclerosis prevents its reversible glutathionylation and may contribute to the impaired vasodilation in atherosclerotic smooth muscle. Analysis of cysteine modifications from atherosclerotic *vs.* normal rabbit aortas showed increased sulfonate formation, corresponding to decreased glutathionylation, reduced NO-induced relaxation and Ca^2+^ reuptake.

The glutathionylation of Ras may also contribute to vascular hypertrophy implicated in atherosclerosis and hypertension in rat vascular smooth muscle cells (VSMCs) [22]. Indeed, the treatment of VSMCs with angiotensin II, which induces vascular hypertrophy, led to glutathionylation and activation of Ras, resulting in increased phosphorylation of p38 and Akt and increased protein synthesis. These effects were dependent upon NADPH oxidase activation and ROS formation [23,24], and were blocked by overexpression of Grx1 or mutation of Ras at the site of glutathionylation (Cys^118^). The glutathionylated Ras may contribute to atherosclerosis by mediating the response to oxLDL in endothelial cells. Indeed, the treatment of bovine aortic endothelial cells with peroxynitrite led to Ras glutathionylation and activation of both ERK and Akt pathways, and some of these observations were recapitulated with oxLDL treatment [25].

A complex relationship exists between protein glutathionylation, Grx and Akt activity within the cardiovascular system [26]. Akt is emerging as a signaling molecule within the heart and vasculature, implicated in various pathological signaling events, as well as in normal development and homeostasis [27]. Deglutathionylation by Grx could participate in regulating the balance between physiological and pathophysiological Akt activation.

An emerging contributor to atherogenesis may further be represented by the tumor necrosis factor-alpha (TNFα), which is thought to induce expression of adhesion molecules on endothelial cells and contribute to vascular smooth muscle cell apoptosis [28]. Pan and Berk [29] treated endothelial cells with a combination of TNFα and cycloheximide and observed Grx activation, pro-caspase-3 deglutathionylation, caspase-3 cleavage and increased apoptosis. This study raises an important question about the potential role of Grx in atheroprotection. However, the role of Grx in cardiovascular disease may not be entirely straightforward, with its roles in disease protection or progression dependent upon cell type, extracellular stimuli, *etc.*

## 3. Tools and Strategies to Monitor Protein *S*-Glutathionylation

*S*-glutathionylation is a redox-dependent post-translational modification with growing relevance in signal transduction. Initially, the meaning of the *S*-glutathionylation was thought to be the protection of cysteine residues against over oxidation to sulfenic (RSOH), sulfinic (RSO_2_H) or sulfonic (RSO_3_H) acids, which can lead to protein inactivation [30]. Later, it was shown that protein glutathionylation directly affects enzyme activities, suggesting a modulator role for this process [2]. At the present time, *S*-glutathionylation is considered a regulatory event in “redox signaling”, and it is involved in several pathways that often cross-talk with each other. The reversibility of this process is a key element to ascribing regulatory, as well as signaling functions to *S*-glutathionylation [31]. The de-glutathionylation may occur via direct thiol/disulfide exchange reactions with GSH, once an appropriate GSH/GSSG ratio has been restored, or by the intervention of glutaredoxin. This enzyme not only deglutathionylates specific proteins, but in the presence of a glutathione-radical generating system, it is also capable of catalyzing *S*-glutathionylation [31].

Glutathionylation and deglutathionylation can also be catalyzed by other enzymes, such as thioredoxin (Trx) and protein disulfide isomerase (PDI). All these enzymes are characterized by the presence of a CXXC motif in the active site where the two cysteines cycle between the reduced form and the oxidized one during the catalytic reaction [32]. Recently, catalysis of de-glutathionylation by sulfiredoxin (Srx), which was previously described as an enzyme that catalyzes the reduction of cysteine-sulfinic acid in peroxiredoxins, was reported [33]. Most recently, a role for the glutathione transferase omega 1 (GSTO1-1) in the glutathionylation cycle was described [34].

**Figure 1:**
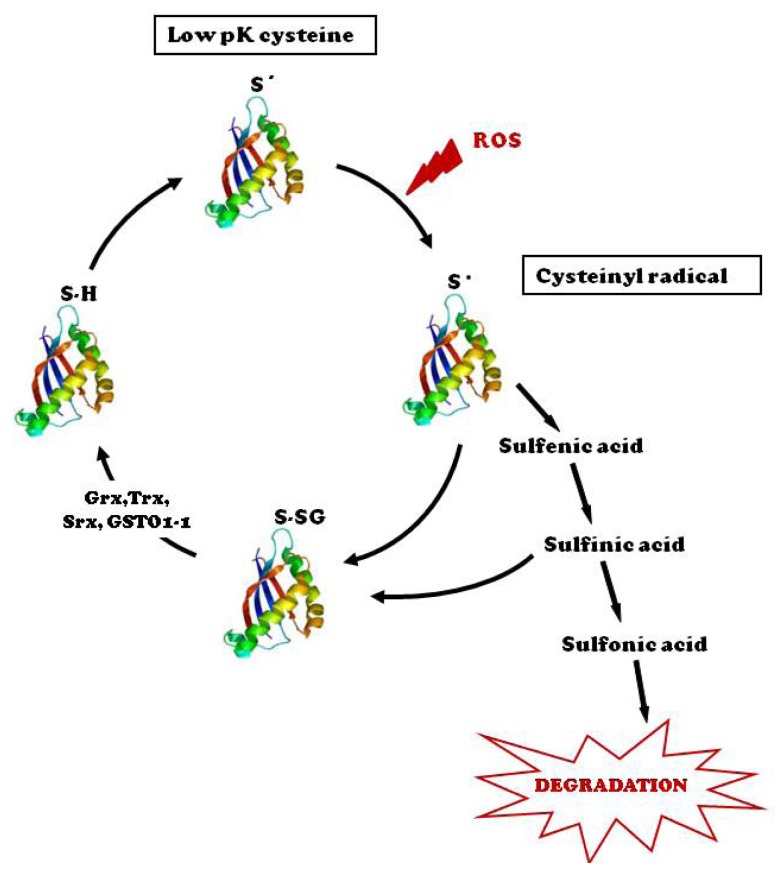
*S*-glutathionylation cycle. Low pKa cysteine residues of proteins are targets for *S*-glutathionylation under oxidative stress conditions. The cysteine residue is first oxidized to form protein sulfenic acids through the formation of a protein cysteinyl radical. Sulfenic acids are highly unstable, undergoing further oxidation to sulfinic and sulfonic acids. Protein sulfonic acids are susceptible to degradation, whereas both cysteinyl radical and sulfenic acid can be conjugated to GSH to form glutathionylated proteins (PSSG). *S*-glutathionylation can be reversed by various enzymes, such as thioredoxin (Trx), glutaredoxin (Grx), protein disulfide isomerase (PDI) and glutathione transferase omega 1 (GSTO1-1).

Taken together, these two process (glutathionylation and deglutathionylation) are defined as the *S*-glutathionylation cycle (Figure 1). Relative to the proteome, although new target proteins appear regularly in the literature, the actual number of *S*-glutathionylated proteins is probably not large and might best be described as the “glutathionome” [35].

Four major types of experimental strategies have been developed to detect protein *S*-glutathionylation. The first method allows the quantification of the total amount of glutathionylated proteins. This technique can be used *in vivo* or *in vitro* and allows quantification of glutathionylated proteins, but is not able to detect glutathione adducts on specific proteins. The second is based on the use of labeled glutathione, by either ^35^S radiolabeling or biotinylation. These techniques can be used *in vivo* or *in vitro* and allow detection of glutathione adducts on *S*-thiolated proteins. The third type is based on the detection of glutathionylated proteins without labeling of the glutathione pool using anti-glutathione antibodies. Both techniques use a bottom-up proteomic approach to identify *S*-glutathionylated proteins. The fourth, and most recent, approach is top-down proteomics, which allows *S*-glutathionylated proteins identification on whole protein extract from cells without the use of labeling or an anti-glutathione antibody [36].

### 3.1. Quantification of Total S-Glutathionylated Proteins

**Figure 2:**
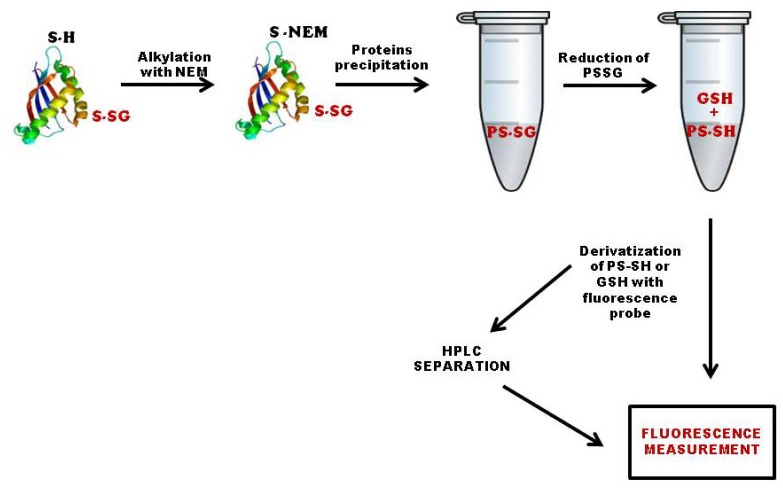
Quantification of total *S*-glutathionylated proteins. The first step of this method is the sample lysis or homogenization in non-reducing buffer containing *N*-ethylmaleimide (NEM) to eliminate free thiols. After this initial step, proteins were precipitated, resuspended and reduced. Protein-sulfhydryls or free glutathione formed after the reduction step was then derivatized with a fluorescence probe, and the fluorescence was measured by fluorometric analysis with or without prior HPLC separation.

A number of methods that allow for quantification of total *S*-glutathionylated proteins either *in vitro* or *in vivo* were described. All methods use sample lysis or homogenization in non-reducing buffer containing *N*-ethylmaleimide to eliminate free thiols, followed by protein precipitation, reduction of the glutathionyl-protein adducts, and derivatization of the protein thiols (PS-SH) or the free GSH, so formed with various fluorescence probes (Figure 2). Finally, fluorescence is measured by fluorometric analysis with or without prior HPLC separation. Using the fluorescence probe, ThioGlo-1™, Townsend and collaborators [37] analyzed the *S*-glutathionylation of liver proteins before and after treatment with the glutathione disulfide mimetic, NOV-002. Most recently, by using monobromobimane as the fluorescence probe and HPLC separation, we found that protein-bound glutathione is increased in lymphocytes of cobalamin c patients and in patients with X-linked adrenoleukodystrophy [38,39]. This method can also be used to monitor antioxidant therapy, as demonstrated recently in children with mitochondrial encephalomyopathies [40]. Using 2,3-naphthalenedicaboxaldehyde as the fluorescence probe and direct fluorescence measurement, Menon and Board [41] quantitatively assayed the total content of *S*-glutathionylated proteins in several mouse tissues treated with the anti-cancer drug, doxorubicin, and in a lymphoblastoid cell line before and after a pro-oxidant stimulus. Their method allows a rapid and simple quantification of changes in the level of protein glutathionylation in response to oxidative stress and drug treatment.

These methods can be useful for the *in vivo* and *in vitro* quantification of total protein glutathionylation, but other methods will have to be employed for the exact identification of single glutathionylated proteins.

### 3.2. Methods Based on Labeling of Glutathione

#### 3.2.1. ^35^S Radiolabeling

**Figure 3:**
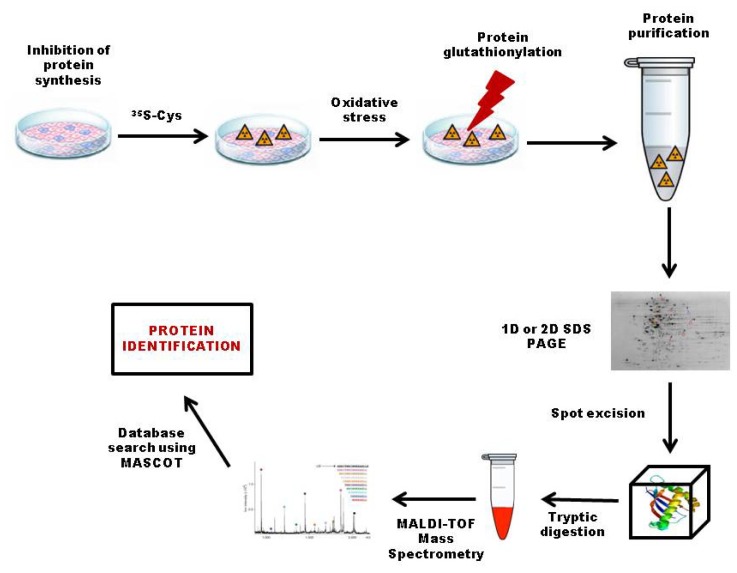
Detection of glutathionylated proteins by ^35^*S*-cysteine labeling. The first step of radiolabeling of the glutathione pool is the inhibition of protein synthesis with cycloheximide, followed by incubation with ^35^*S*-cysteine. ^35^*S*-glutathione is then synthesized by the glutathione cycle. After the initial labeling step, the cells are placed under conditions of oxidative stress, leading to protein *S*-thiolation. Proteins are then extracted and separated on non-reducing mono- or bi-dimensional sodium dodecyl sulfate polyacrylamide gel electrophoresis. *S*-thiolated proteins can be visualized by autoradiography, and the radioactive gel spot are excised and digested with trypsin. The resultant tryptic peptides are subjected to matrix-assisted laser desorption/ionization (MALDI-TOF) followed by database searching to identify *S*-glutathionylated proteins subjected to bottom-up proteomic analysis in order to identify *S*-glutathionylated proteins.

Radiolabeled glutathione is a convenient tool for the analysis of protein glutathionylation, which allows a very sensitive and quantitative detection of glutathionylated proteins. *In vitro*, the glutathionylation of several proteins has been analyzed using ^35^*S*-GSH [42–45]. Radiolabeling of the glutathione pool by ^35^*S*-cysteine has been the most widely used method for proteomic analysis of glutathionylated proteins *in vivo* (Figure 3). The first step consists of inhibition of protein synthesis with cycloheximide, followed by incubation in the presence of ^35^*S*-cysteine. After the labeling step, cells are placed under oxidative stress conditions, by the addition of oxidants, such as H_2_O_2_ and diamide, or NO donors, such as nitrosoglutathione (GSNO) or PABA/NO. Alternatively, *S*-thiolation is obtained by increasing ROS production, for example, by induction of the respiratory burst in monocytes, which leads to *S*-thiolation of several proteins [46–48]. After this second step, proteins can be extracted and separated on non-reducing mono- or bi-dimensional gels. *S*-thiolated proteins can be visualized after gel drying by autoradiography or phosphor imaging technologies. DTT treatments of radioactive samples should lead to a loss of the radioactive signal, thereby confirming that the labeling is linked to *S*-thiolation. With the development of proteomic approaches, this method was further adapted for large-scale identification of *S*-thiolated proteins using 2D gels and peptide mass fingerprinting. The first study was performed on human T lymphocytes, where ^38^*S*-thiolated proteins could be identified after treatment with diamide or H_2_O_2_[49]. Subsequently, similar studies allowed identification of a number of *S*-thiolated proteins in several cell types and organisms. The ^35^*S*-cysteine labeling method has, however, several major drawbacks. The major problem resides in the necessary pretreatment with protein synthesis inhibitors that could perturb cell physiology. Moreover, this method does not allow for discrimination between the different possible types of *S*-thiolation, although glutathionylation is considered to be predominant. Indeed, some of the radiolabeled proteins might not be glutathionylated, but cysteinylated or *S*-thiolated by other low-molecular-weight compounds synthesized from cysteine. The contribution of glutathionylation to the total labeling can be estimated in the presence of buthionine sulfoximine (BSO), a specific inhibitor of the glutathione synthesis. In human T-cells, more than 80% of the radiolabeling is lost in the presence of BSO, thereby confirming that glutathionylation is the major type of *S*-thiolation [49]. Another limitation is linked to the necessity to perform 2D gels to visualize the *S*-thiolated proteins. The loading limit of 2D gels allows only the identification of abundant proteins. Though this problem might be partly overcome by fractionation of the extract before 2D electrophoresis, low abundance proteins will probably not be identified with this method. Similarly, proteins with a high or low pI or a very high or very low molecular weight will not be detected. The sensitivity of this method is also limited by the low specific activity of the ^35^*S*-labeled glutathione pool. Another drawback is that the use of this method is restricted to cell cultures, thereby avoiding studies on whole organisms under physiological conditions and strongly limiting genetic analyses. Finally, this method can only detect proteins undergoing glutathionylation during the treatment, while some proteins might be already glutathionylated under basal conditions.

Despite its numerous limitations and drawbacks, the ^35^Scysteine labeling method has allowed identification of most known *S*-thiolated proteins. The method can be useful for identification of most abundant *S*-thiolated proteins in cell cultures, while other methods will have to be employed for the analysis of low abundance proteins.

#### 3.2.2. Biotinylated Glutathione

**Figure 4:**
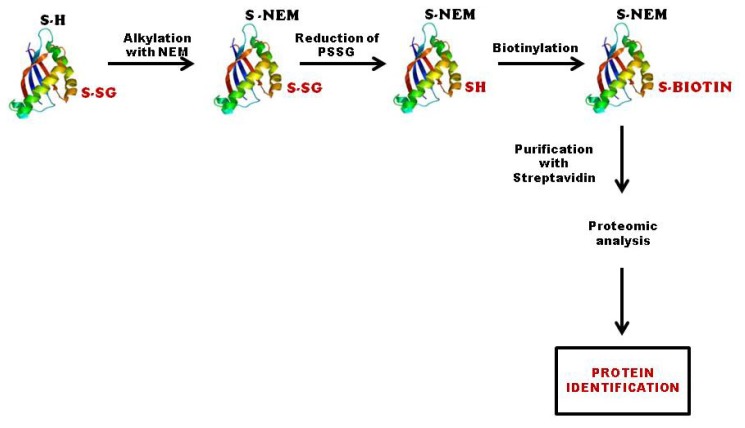
Detection of glutathionylated proteins by biotin labeling. Proteins are first alkylated with *N*-ethylmaleimide to block free sulfhydryls. Oxidized sulfhydryls are then reduced with Grx3 and labeled with biotin. Biotin-labeled proteins are affinity purified on avidin-conjugated agarose beads. After extensive washing with detergent buffer, proteins bound to avidin via a mixed disulfide bond with biotinylated glutathione can be eluted by incubation with reducing agents (DTT or β-mercaptoethanol), separated on two-dimensional sodium dodecyl sulfate polyacrylamide gel electrophoresis (2D SDS PAGE) and subjected to proteomic analysis.

Biotinylated glutathione can be easily synthesized *in vitro* either in the reduced (BioGSH) or in the oxidized (BioGSSG) forms. The synthesis of these compounds is based on the use of a water-soluble biotinylation reagent (sulfosuccinimidyl-6-(biotinamido)-hexanoate, sulfo-NHS-biotin). This reagent is used to couple biotin to the primary amino groups of glutathione under mild alkaline conditions using an amine-free buffer. After the reaction is completed, any remaining biotinylation reagent is quenched by the addition of an amine-containing buffer to a 10-fold molar excess of the starting sulfo-NHS-biotin concentration. The biotinylation reagent is reacted with the reduced form (GSH) at a 1:1 molar ratio, while a 2:1 molar ratio is used with the oxidized form. However, when the biotinylated reagent is coupled to reduce glutathione, it can react with the sulfur atom of glutathione, decreasing the total amount of free-thiol biotinylated glutathione. In the case of oxidized glutathione, the presence of two primary amino groups at the opposite ends of GSSG leads to the incorporation of two biotin moieties into the GSSG molecule. The resulting biotinylated glutathione molecules can be used as an effective marker for oxidant induced *S*-glutathionylation. The presence of the biotin moiety on glutathione allows for a sensitive and specific detection of glutathionylated proteins by non-reducing Western blot probed with commercially available streptavidin-horseradish peroxidase or anti-biotin antibodies. When BioGSSG is used, it mimics a defined component of oxidative stress, namely, a shift in the glutathione redox couple to the oxidized disulfide state. By contrast, BioGSH does not induce oxidative stress, and it is used in combination with oxidants, such as diamide or H_2_O_2_. Biotin-labeled proteins can be affinity purified on avidin-conjugated agarose beads. After extensive washing with detergent buffer, proteins bound to avidin via a mixed disulfide bond with biotinylated glutathione can be eluted by incubation with reducing agents (DTT or β-mercaptoethanol) and identified by mass spectrometry (Figure 4). BioGSSG allowed identification of 11 glutathionylated proteins by LC-MS/MS in rat heart during post-ischemic reperfusion [50].

Biotin-based strategies for proteomic analysis of glutathionylated proteins have several advantages compared to ^35^*S*-cysteine labeling methods. First, protein synthesis does not have to be inhibited during the oxidative stress treatment. Second, this method only detects glutathionylated proteins rather than all *S*-thiolation targets. Third, the affinity purification is very specific and overcomes 2D gel limitations. The targets can be analyzed on 2D gels only loaded with glutathionylated proteins rather than total extracts, allowing detection of significantly less abundant proteins. Alternatively, eluted proteins can be analyzed by highly sensitive and high throughput proteomic methods, such as nanoLC-MS/MS. Finally, the presence of the biotin tag on the proteins of interest allows their detection by multiple methods, such as immunoblotting with or without prior immunoprecipitation using biotin antibodies or horseradish peroxidase-conjugated avidin (HRP-avidin) [51], batch or column-based affinity purifications or cellular localization by fluorescence microscopy [50]. The major drawback of the methods based on biotinylated glutathione is the presence of the bulky biotin tag on the glutathione molecule that might perturb the function of proteins interacting with glutathione and especially those controlling glutathionylation. A common drawback of both labeling methods is that they do not give access to proteins glutathionylated under basal conditions.

### 3.3. Methods Utilizing Anti-Glutathione Antibodies

Glutathionylated proteins can also be detected with commercially available anti-glutathione antibodies. Methods based on such antibodies are promising, since they could overcome most problems encountered with ^35^S and biotin labeling methods. Indeed, with anti-glutathione antibodies, glutathionylated proteins could be analyzed under more physiological conditions, since no pretreatment is required. This could allow detection of glutathionylated proteins by Western blots with 1D or 2D gels, by immunoprecipitation or even by immunocytolocalization. Almost all published studies have been performed with a mouse monoclonal antibody (Virogen, Watertown, MA, USA), which has proven useful to analyze, *in vivo*, the glutathionylation of actin [52–57], myosin [57,58], tubulin [56,59,60], HSP70 [61,62], neurofilaments [56] and Type 1 calcium release channels [63,64]. These proteins can probably be detected in total extracts, because they are very abundant. Indeed, this antibody exhibits a low sensitivity that greatly limits the number of glutathionylated proteins detected. This sensitivity issue can be partly overcome by working on purified proteins or fractions enriched with the protein of interest, even if this preliminary enrichment is only possible when the target protein is known, and it is therefore not applicable for proteomic identification of unknown glutathionylated proteins. In total extracts, the anti-glutathione antibody only detects a few abundant proteins [50]. Every proteomic study based on the use of this antibody led to the identification of only four or five abundant proteins, such as HSP70 or actin [62,65,66]. The number of glutathionylated proteins detected appears higher with strong inducers of glutathionylation, such as PABA/NO [33,67]. A major drawback of the anti-glutathione antibody concerns its specificity. Indeed, glutathione is a very flexible molecule that can potentially exhibit hundreds of conformations, either in solution or bound to proteins [68]. Hence, the affinity of the antibody for glutathionylated proteins is likely to vary greatly, depending on the conformation of the glutathione adduct and the environment of the thiolated cysteine. Overall, the anti-glutathione antibodies currently available can prove useful for the analysis of individual proteins, but do not appear to be appropriate for large-scale detection of glutathionylated proteins by proteomic approaches.

### 3.4. Top-Down Proteomics Approach

Most recently, a top-down proteomics approach was used to identify protein *S*-glutathionylation and *S*-cysteinylation in *Salmonella typhimurium* in response to infection-like conditions [36]. Liquid chromatography-coupled mass spectrometry (LC-MS)-based top-down proteomics is an emerging method in which whole/intact proteins are separated and fragmented directly into the mass spectrometer to achieve both protein identification and characterization. This is in contrast to the well-established bottom-up LC-MS approach in which proteins are digested with a protease into smaller peptides and analyzed by tandem mass spectrometry to give protein identity from peptide level information. Bottom-up proteomics is still a good method given its ability to identify large numbers of proteins, but is not able to distinguish proteoforms [69], which constitute the functional proteome essential to understand biological systems. With this approach, *S*-glutathionylated proteins could be identified in biological systems without using any labeling procedures, thus allowing sensitive and specific access to proteins glutathionylated under basal conditions.

### 3.5. Other Methods

Several other methods have been proposed to detect and identify a numbers of glutathionylated proteins. Some studies have used immobilized glutathione or its analogs to affinity purify proteins containing reactive cysteines susceptible of undergoing glutathionylation. While these methods do not allow *in vivo* analyses, they may be complementary to techniques based on radiolabeled or biotinylated glutathione for the analysis of glutathionylation targets *in vitro*. GSH and GSSG affinity matrices allowed for identification of seven candidate glutathionylated proteins by Western blot [70] and were also used to study the glutathionylation of SERCA [21] and p53 [71]. Liquid chromatography/electrospray ionization mass spectrometry (ESI-MS) [72] or cation exchange HPLC coupled with spectrophotometric detection [73] were used for the quantification of erythrocytes glutathionyl-hemoglobin (GS-Hb) content.

Recently, a bioinformatics framework to predict *S*-glutathionylation sites by employing machine learning methods based on protein sequences was reported [74]. All the *S*-glutathionylation proteins and their corresponding modification sites are manually collected from the literature, so that a series of classifiers are built to predict *S*-glutathionylation sites based on support vector machines (SVMs). Different features are extracted from protein sequences for prediction of *S*-glutathionylation sites. Results obtained in five-fold cross-validation demonstrate the effectiveness of this method, with a Receiver Operating Characteristic curve _(_AUC) score of 0.879. This method could provide putative *S*-glutathionylation sites for future experimental verification.

## 4. Physiological Effects of Protein Glutathionylation

### 4.1. Myocardial Contraction

#### 4.1.1. Ryanodine Receptor

Calcium release through cardiac ryanodine receptors (RyR_2_) triggers heart muscle contraction. RyR_2_ is a large ligand-activated intracellular Ca^2+^ release channel located at the endoplasmic and sarcoplasmic reticulum (SR). In the myocardium, RyR_2_ plays a crucial role in mediating excitation contraction coupling by increasing intracellular Ca^2+^ after an action potential. It is regulated by direct phosphorylation at different sites by cAMP-dependent kinase (PKA) or calmodulin kinase II (CaMKII). In addition to phosphorylation, direct oxidation has also been implicated as a regulator of the channel’s function. This is not surprising, considering that this tetrameric channel contains 364 cysteines with about 84 of them having free thiol groups. Basal *S*-glutathionylation of RyR_2_ was discovered in microsomal fractions enriched in SR vesicles isolated from dog cardiac ventricular muscle [75]. Under physiological conditions, such as tachycardia and exercise, RyR_2_*S*-glutathionylation increases, and this suggests that cardiac cells utilize this redox modification to increase RyR_2_ activity when the demand is increased [76]. The RyR2 activity is enhanced also in heart failure, and this presumably contributes toward decreasing calcium content in sarcoplasmic reticulum and inducing calcium release abnormalities observed in heart failure. The number and identity of reactive cysteines in the RyR_2_ are presently unknown, like the physiological sources of ROS responsible for RyR_2_ redox modifications. With both exercise and tachycardia, the administration of the NADPH oxidase (NOX) inhibitor, apocynin, prevented RyR_2_*S*-glutathionylation and attenuated Ca^2+^ release from the SR. However, it is important to consider that apocynin may not be a specific NADPH inhibitor, but rather, an antioxidant, which could also explain its ability to decrease RyR_2_*S*-glutathionylation in these models [77]. The redox state of RyR_2_ is altered in heart failure, leading to enhanced RyR_2_ activity, which presumably contributes to decrease SR calcium content and induce other calcium release abnormalities observed in heart failure. Therefore, greater understanding of RyR_2_ redox modulation is necessary to counteract the deleterious consequences of RyR_2_ activity deregulation caused by oxidative stress [78].

#### 4.1.2. Sarco/Endoplasmic Reticulum Ca^2+^ ATPase (SERCA)

Of the three mammalian members belonging to the sarco(endo)plasmic reticulum Ca^2+^ ATPase (SERCA) family, SERCA2 is evolutionarily the oldest and shows the most wide tissue-expression pattern [79]. Two major SERCA2 splice variants are well characterized: the muscle-specific isoform, SERCA2a, and the housekeeping isoform, SERCA2b. SERCA2b is found in the ER of most cell types and is considered the housekeeping isoform. The muscle-specific isoform, SERCA2a, is expressed in the sarcoplasmic reticulum (SR) of the heart and slow-twitch skeletal muscle. Several interacting proteins and post-translational modifications of SERCA2 were identified, which may modulate the activity of the Ca^2+^ pump. SERCA2, the dominant isoform in the heart and vasculature, acts as an inward pump that utilizes the energy from ATP to drive the removal of intracellular free Ca^2+^ into the SR Ca^2+^ store [80]. The ability of this pump to maintain cytosolic free Ca^2+^ and the quantity available in the SR store during systole makes SERCA2 a major determinant of cardiac contractility and smooth muscle tone. The activity of SERCA2 is regulated by the accessory membrane protein, phospholamban (PLN). PLN binds to and inhibits SERCA2 when dephosphorylated. The phosphorylation of PLN by PKA at Ser-16 or calmodulin-dependent protein kinase II (CaMKII) at Thr-17 dissociates PLN from SERCA and relieves its inhibitory effect on SERCA activity. In addition, direct *S*-glutathionylation can modulate the SERCA2’s activity, and this occurs on the reactive thiol of Cys^674^[81]. Notably, under physiological conditions in arterial smooth muscle, endothelium-derived NO combines with the superoxide radical to form the highly reactive peroxynitrite (ONOO−). This then reacts with cytosolic glutathione and reversibly glutathionylates a number of free SH groups in SERCA, of which the one in Cys^674^ is pivotal in activating SERCA2 [21].

In intact cells or arteries, *S*-glutathionylation of the pump by NO (in the form of its active “effector” peroxynitrite) results in accelerated Ca^2+^ uptake and in relaxing of vascular smooth muscle. In atherosclerotic aorta, NO does not increase *S*-glutathionylation of SERCA, because of the irreversible oxidation of Cys^674^ to sulfonic acid, and as a result, fails to stimulate its activity [21,81]. *S*-glutathionylation affects Ca^2+^ homeostasis also in cultured aortic endothelial cells, where diamide promoted Ca^2+^ release from inositol 1,4,5-trisphosphate (IP_3_)-sensitive internal Ca^2+^ stores and elevated basal free cytosolic Ca^2+^ concentration. This effect was related to the glutathionylation of the IP_3_ receptor and the plasmalemmal Ca^2+^ ATPase pump [82].

In a rabbit model of atherosclerosis, SERCA *S*-glutathionylation in abdominal aorta was increased, and a decreased relaxation was observed; thus, the loss in vessel relaxation in the aorta of rabbits with atherosclerosis could be explained by inhibition of *S*-glutathionylation [21]. In the heart, stimulation of SERCA activity and cardiomyocyte contractility by the thiolating nitroxyl anion generator, Angeli’s salt, is also accompanied by *S*-glutathionylation and stimulation of SERCA activity [83].

#### 4.1.3. The Endothelial Nitric Oxide Synthase (eNOS)

The endothelial nitric oxide synthase (eNOS) is constitutively expressed and is involved in regulating normal cellular function by converting l-arginine to l-citrulline. eNOS generates nitric oxide (NO), which has different roles in the cardiovascular system, including the control of blood pressure and smooth muscle tone, regulation of platelet aggregation, development of arteriosclerosis, cytoprotection and cytotoxicity [3]. eNOS can be modulated by direct *S*-glutathionylation [84]. The *S*-glutathionylation of eNOS decreases its activity and increases O^2−^ formation, as measured using paramagnetic spin trapping. Cys^689^ and Cys^908^ in the reductase domain have been identified as the sites of oxidation by GSSG using liquid chromatography-tandem mass spectrometry, and molecular modeling around the sites of *S*-glutathionylation revealed a dysfunctional alignment between the FAD and FMN binding domains that decreases the electron transfer between flavins and improves access to oxygen, then converts it to O^2−^. The mutation of these cysteine residues had little effect on eNOS activity, but attenuated O^2−^ formation after treatment with GSSG, thus demonstrating that the two cysteines are key redox sites in eNOS activity regulation. Under pathological oxidative stress, *S*-glutathionylation of eNOS can cause a loss in endothelium-dependent relaxation, leading to hypertension [84,85]. This redox mechanism of eNOS modulation was found to occur in hypertensive rats, which had a normal response to an NO-donor, but had a large deficit in acetylcholine-mediated relaxation compared to control animals. Interestingly, in the aorta of these rats, there was increased eNOS *S*-glutathionylation, and the relaxation abnormalities were rectified by the addition of a thiol-specific reducing agent (DTT) to isolated vessels. These results suggest that under pathological oxidative stress, *S*-glutathionylation of eNOS can cause a loss in endothelium-dependent relaxation, leading to hypertension.

#### 4.1.4. Na^+^-K^+^ ATPase

The cardiac Na^+^-K^+^ pump is a heterodimeric membrane protein composed of a 100-kDa α subunit (consisting of 10 transmembrane segments) and a glycosylated 55-kDa type II membrane protein, the β subunit [86,87]. The active transport mediated by this ubiquitous ATP-dependent pump maintains the electrochemical gradients for Na^+^ and K^+^ across cell membranes in all tissues and is estimated to consume up to 20%–30% of ATP under resting conditions [88]. However, the electrochemical energy of the Na^+^ gradient also serves in “secondary” co- and counter-transport of other ions and of important organic compounds with broad implications for cellular physiology and pathophysiology [86]. In heart, the secondary active transport of Ca^2+^ has received particular attention for its role in excitation-contraction coupling. Raised intracellular Na^+^ levels play a key role in the Ca^2+^-dependent contractile abnormalities and arrhythmias characterizing heart failure [89,90]. Indeed, a small increase in Na^+^ causes an increase in Ca^2+^ and enhances contractility under physiological conditions. In the presence of larger increases of intracellular Na^+^ and Ca^2+^, Ca^2+^ is spontaneously released from the sarcoplasmic reticulum (SR) during diastole. Therefore, understanding the mechanisms of pump regulation is critical and may be very useful in treatments [91].

The β1 subunit of the Na^+^-K^+^ pump is a substrate for glutathionylation that occurs at cysteine 46, causing pump inhibition. This β_1_ subunit contains seven cysteine residues. Six of these, located in the extracellular domain, are linked by three disulfide bonds [92]. Only one cysteine, Cys-46, has a free sulfhydryl group and is a good candidate for glutathionylation. Mutagenesis of α_1_/β_1_ Na^+^-K^+^ pump heterodimers, expressed in *Xenopus* oocytes, confirmed that Cys-46 was reactive and established that glutathionylation of it was causally related to Na^+^-K^+^ pump inhibition. The β_2_ and β_3_ subunits have only six cysteine residues linked by disulfide bonds; thus, β_2_/β_3_ heterodimers are not sensitive to oxidation-induced inhibition [93]. Liu *et al.* [94] have recently examined whether glutathionylation depends on the conformational changes in the Na^+^-K^+^ pump cycle. Indeed, the crystal structure indicates that the side chain of cysteine 46 is exposed to the lipid bulk phase of the membrane and not accessible to the cytosolic glutathione [95,96]. However, the Na^+^-K^+^ pump undergoes large changes in molecular structure during its catalytic cycle [97]. The Na^+^-K^+^ pump undergoes changes between states, referred to as E1 and E2 conformations, during its pumping cycle, with substantial movement of β subunits relative to α subunits [98]. This conformational change from E2 to E1 may shift the Cys-46 of the β1 subunits into a cytosolic domain, facilitating glutathionylation. Liu *et al.* [94] have measured β_1_ subunit glutathionylation in membrane fragments and in ventricular myocytes. Large signals for glutathionylation were detected with the biotin-GSH and GSH antibody techniques when membrane fragments were suspended in a solution that stabilizes the E1ATP conformation of the Na^+^-K^+^-ATPase, whereas the signal was much smaller in a solution stabilizing the E2 conformation. No difference in glutathionylation between the E2 and K-bound E2 was observed. The exposure of myocytes to ouabain decreased glutathionylation, and it was consistent with the shift from E1 toward E2 conformations [98]. However Grx1, which mediates deglutathionylation, co-immunoprecipitates with β_1_ subunits in myocytes lysate and may abolish oxidant induced Na^+^-K^+^ pump inhibition [93]. The increased interaction of Grx1 with the β_1_ subunit in E1 compared with E2 states may reflect a mechanism that prevents progressive amplification of E1-dependent β_1_ subunit glutathionylation and an excessive pump inhibition.

Because of the critical role of β_1_ glutathionylation in the inhibition of the Na^+^-K^+^ pump in the heart, Liu *et al.* [99] have recently investigated the oxidative inhibition of the vascular Na^+^-K^+^ pump via NADPH oxidase-dependent β_1_ glutathionylation. The authors show that Ang II inhibits the vascular pump by increasing β_1_ glutathionylation, and this is mediated by the activation of NADPH oxidase. Thus, redox-dependent pump inhibition may be significant also in the regulation of vascular tone.

#### 4.1.5. Contractile Proteins

##### 4.1.5.1. Myosin

Myosin is sensitive to *in vitro* glutathionylation, and three potential sites of glutathione binding have been identified by MALDI-TOF analysis, two of them located on the myosin head [58]. Glutathionylation of myosin has an important impact on the protein structure, as documented by the lower fluorescence quantum yield of glutathionylated myosin and its increased susceptibility to the proteolytic cleavage. Myosin function is also sensitive to glutathionylation, which modulates its ATPase activity, depending on GSSG redox balance. Thus, like the phosphorylation/dephosphorylation cycle, glutathionylation may represent a mechanism by which glutathione modulates sarcomere functions depending on the tissue redox state, and myosin may constitute a muscle redox-sensor.

##### 4.1.5.2. α*-*Actin

α-actin is also particularly sensitive to binding of glutathione in isolated cardiac and skeletal myofibrils under conditions of oxidative stress [57]. The glutathionylation of α-cardiac actin occurs non-enzymatically, but via spontaneous oxidation of a cysteinyl residue to a cysteinyl sulfenic acid intermediary, a mechanism previously reported by Johansson and Lundberg for cytoskeletal β-actin [100] and by Dalle-Donne *et al.* [101] for rabbit skeletal muscle actin. By light scattering, it has been demonstrated that α-cardiac actin polymerized slower than the native protein, when glutathionylated *in vitro*. Thus, as for cytoskeletal β-actin [49,52,55] and for skeletal muscle actin [101], even β-actin could constitute a direct target for oxidative modification in human heart, and its glutathionylation may represent a mechanism by which glutathione can modulate sarcomere functions, depending on the redox state of the tissue.

In multiple models, including ischemia-reperfusion, increased oxidative stress results in the glutathionylation of sarcomeric actin. Pizarro and Ogut [102] examined the functional impact of glutathionylated actin on the interaction with myosin-S1. They found that substituting glutathionylated F-actin for unmodified F-actin reduced the maximum actomyosin-S1 ATPase, and this was accompanied by an increase in the activation energy of the steady-state ATPase. Measurement of steady-state binding did not suggest a large impact of actin glutathionylation on the binding to myosin-S1. However, transient binding and dissociation kinetics determined by stopped-flow methods demonstrated that although actin glutathionylation did not significantly alter the rate constant of myosin-S1 binding, there was a significant decrease in the rate of ATP-induced myosin-S1 detachment in the presence of ADP. These results suggest that actin glutathionylation may play a limited, but defined, role in the alteration of contractility following oxidative stress to the myocardium, particularly through a decrease in the actomyosin ATPase activity.

##### 4.1.5.3. Myosin Binding Protein C (MyBP-C)

Recently, Lovelock *et al.* [103] have reported a significant increase in the glutathionylation of cardiac myosin binding protein-C (MyBP-C) in animals with diastolic dysfunction. Their findings support the hypothesis that changes in cross-bridge kinetics correlate with MyBP-C *S*-glutathionylation and that this oxidative modification may be responsible for the changes in cardiac dynamics. MyBP-C may directly interact with actin in the thin filaments, resulting in increased cross-bridge kinetics [104]. Whatever the case, the modification by *S*-glutathionylation of one or more cysteine residues of MyBP-C likely alters the proximity of the cross-bridges to or their interactions with the thin filament.

##### 4.1.5.4. Troponin

Troponin I (TnI) in mammalian fast-twitch (type II) fibers can be readily *S*-glutathionylated, and this results in a large increase in the Ca^2+^ sensitivity of the contractile apparatus with no detectable change in maximum force [105]. Mammalian TnIf has three cysteine residues, but when TnIf is in the troponin complex with troponin C and troponin T, only Cys^133^ is accessible and reactive [106]. Thus, the *S*-glutathionylation of TnIf is only attributable to a link with Cys^133^. Noteworthy, Mollica *et al.* [105] found that moderate-intensity cycling exercise for 40 min led to an approximately four-fold increase in the *S*-glutathionylation of TnIf in the vastus lateralis muscle of human subjects. Given that *S*-glutathionylation of TnIf of human type II fibers was found, like in rat type II fibers, to result in a large increase in Ca^2+^ sensitivity, the authors concluded that *S*-glutathionylation of TnIf may influence muscle performance in exercising humans. Increasing the Ca^2+^ sensitivity of the contractile apparatus by glutathionylation could be of great benefit in countering factors occurring with normal exercise and causing muscle fatigue [107].

In addition to increase contractile Ca^2+^ sensitivity, *S*-Glutathionylation of TnIf also increases the peak twitch force and rate of force development to action potential stimulation [108]. Thus, the overall effects of the *S*-glutathionylation of TnIf are highly comparable to those occurring with the phosphorylation of myosin light chain 2 [109], with both causing similar sized increases in Ca^2+^ sensitivity and similar twitch force potentiation in fast-twitch muscle fibers, though by distinctly different mechanisms. However, if the activity continued for too long, other deleterious effects of the oxidants could antagonize and counter the potentiating effects of the *S*-glutathionylation of TnIf [110,111].

### 4.2. Myocardial Metabolism

#### 4.2.1. Complex I (CI)

A wide range of mitochondrial membrane proteins contain exposed thiols that react with GSSG to form mixed disulfides, but a prominent target seems to be the respiratory chain enzyme, Complex I (CI) [13,112–114]. In isolated cardiac mitochondria, CI is highly susceptible to glutathionylation under conditions of oxidative stress, showing a dose- and time-dependent inactivation after treatment with GSSG [115]. Furthermore, in cultured cardiomyocytes, CI activity was strongly inhibited after *in vivo* treatment with hydrogen peroxide [115]. CI represents the entry point of electrons into oxidative phosphorylation, making this enzyme particularly critical for cellular health [116]. CI glutathionylation leads to an increase of superoxide production, and, following de-glutathionylation, superoxide returns to basal levels. Within intact mitochondria, most of this superoxide produced after CI glutathionylation is converted to H_2_O_2_, which can then diffuse into the cytoplasm. By this mechanism, mitochondria may regulate redox signaling, and glutathione oxidation contributes to the pathological changes occurring during oxidative stress [13].

#### 4.2.2. Complex II (CII)

In addition to CI, Chen *et al.* [9] have demonstrated that myocardial infarction leads to *S*-glutathionylation of Complex II (CII), confirmed *ex vivo* using a Langendorff system of ischemia and reperfusion. LC-MS/MS demonstrated *S*-glutathionylation on Cys-90 of purified CII. This modification enhances enzyme activity and reduces O^2−^ production [9].

Mitochondrial targets for glutathionylation continue to accumulate, although most mitochondrial proteins are glutathionylated only transiently, and a smaller fraction is persistently glutathionylated [114]. CII requires a physiological glutathionylation to explicate its optimal function, thus representing an example of a protein that is persistently glutathionylated [9,117].

#### 4.2.3. ATP synthase Complex (CV)

Furthermore, the mitochondrial ATP synthase complex undergoes a number of cysteine-specific oxidative modifications in the failing heart [118]. In particular, the authors found that the α-subunit of the ATP synthase Complex (CV) is glutathionylated in mitochondria isolated from dyssynchronous heart failure (DHF) and could be induced by GSSG in a dose-dependent manner. Interestingly, the levels of glutathionylated CV were partially normalized in cardiac resynchronization therapy (CRT), an effective clinical treatment for heart failure patients with conduction delay, impaired contraction and energetics.

#### 4.2.4. Sirtuin-1

Sirtuins are a highly conserved family of histone/protein deacetylases and have been shown to participate in biological functions related to the development of heart failure, including regulation of energy production, oxidative stress, intracellular signaling, angiogenesis, autophagy and cell death/survival. Mammals possess seven sirtuins. Sirtuin 1 (Sirt-1) is an NAD^+^-dependent deacetylase located in the nucleus. In the vascular endothelium, Sirt-1 promotes cellular homeostasis through deacetylating a wide variety of targets, including eNOS, forkhead box transcription factor O1 (FOXO1) and p53 [119]. Sirt-1 activity depends on the availability of NAD^+^, such that increases in the [NAD^+^]/[NADH] ratio during caloric restriction activate the enzyme and can regulate gene expression and apoptosis. Using the purified protein, Zee *et al.* [120] demonstrated that nitrosoglutathione (GSNO) decreased Sirt-1 activity, and this effect was mediated by *S*-glutathionylation. The authors identified Cys^67^ as the site of *S*-glutathionylation by Linear Ion Trap-Orbitrap MS [120]. In addition, also Cys^482^ has been found to be modified by hydroxynonenal on MALDI-TOF-TOF analysis [121].

#### 4.2.5. Aldose Reductase

Aldose reductase (alditol: NADP^+^ oxidoreductase, AR) is an aldehyde metabolizing enzyme that catalyzes the NADPH-dependent reduction of aldo sugars and a variety of aldehydes. Aldose reductase is an NADPH-dependent monomeric protein that belongs to the aldo-keto reductase superfamily [122,123]. The protein is expressed basally in many tissues, and its expression is enhanced by growth factors, such as fibroblast growth factor and platelet-derived growth factor [124], and by cytokines, such as tumor necrosis factor-α [125,126]. The enzyme is susceptible to covalent modification by several disulfides and thiol agents at Cys^298^[127–129]. However, only GSSG was able to determine inactivation of the enzyme [130]. Its inhibition has been shown to increase oxidative injury and to abolish the late phase of ischemic preconditioning.

In the vasculature, aldose reductase mediates high-glucose-induced smooth muscle cell proliferation, and in cardiac tissue, it can modulate the late phase of ischemia reperfusion [131]. Rat hearts subjected to ischemia, *in situ* or *ex vivo*, display a 2–4-fold increase in AR activity. The AR activity was not further enhanced by reperfusion. *In vivo*, hearts subjected to ischemia reperfusion showed a two- to four-fold increase in aldose reductase activity, demonstrating that reactive oxygen species formed in the ischemic heart activate AR, and this occurred by modifying its cysteine residues to sulfenic acids. By MALDI-TOF MS, it has been found that aldose reductase was *S*-glutathionylated at Cys^298^ and Cys^303^[132,133]. The role of AR in myocardial metabolism during ischemia needs to be investigated in greater detail to fully assess the functional significance of the activation of this enzyme by redox modification of its cysteine residues.

#### 4.2.6. Hemoglobin

The binding of glutathione to the Cys^93^ of the hemoglobin (Hb) beta chain has been known for more than 20 years and is associated with inhibition of Hb *S*-polymerization, increased oxygen affinity and a reduced alkaline Bohr effect [134,135]. At least three different types of glutathionylated Hb can exist in erythrocytes: a mixed disulfide bond between GSH and normal Hb; a disulfide bond between the Cys^93^ of the metHb beta chain and GSSG; and a disulfide bond between the other cysteine residues of metHb alpha chain and/or metHb beta chain and GSSG [136,137]. Recently, Metere *et al.* [138] found that, besides Cys^93^, Hb glutathionylation occurs also at Cys^112^ of the β-chain, providing a new potential GSH source hitherto unknown. They found that CO treatment of whole blood increases the GSH concentration in red blood cells cytosol, and this is linked to a significant Hb deglutathionylation. This process does not activate glycolytic metabolism, boosts the pentose phosphate pathway, increases glutathione reductase activity and decreases GSSG concentration, thus evidencing a CO signaling in human RBC driven by Hb glutathionylation. Hb is also a target for *S*-nitrosylation [139], and this modification may play a role in blood flow regulation [140,141]. Overall, these evidences made Hb a very useful marker of oxidative stress [142] and a good candidate in monitoring redox aspects of cardiovascular diseases. However, so far, neither the modification of Hb by glutathione nor by NO has been translated into effective treatments in such diseases.

### 4.3. Myocardial Proliferation and Hypertrophy

#### p21Ras

p21Ras is one major regulator in growth factor signaling in the vasculature. It is known to have four exposed redox-sensitive cysteines [143,144]. Adachi *et al.* [22] have demonstrated that angiotensin II-induced hypertrophy in vascular smooth muscle cells is regulated by the *S*-glutathionylation of p21Ras at Cys^118^. The GSH-adduct and the resulting activation of the p21Ras-MEK-ERK pathway were reversed by overexpression of catalase, glutaredoxin-1 or dominant negative p47Phox. In cardiac tissue, thioredoxin regulates pressure-overload-induced cardiac hypertrophy. Compared with wild-type animals, those without thioredoxin had greater cardiac hypertrophy, while overexpression of the protein resulted in attenuated cardiac size. The mechanism appeared to be due to modulation of p21Ras at Cys^118^, as demonstrated by *in vitro* models of strain and alpha-adrenergic receptor-stimulated cardiac hypertrophy in cardiomyocytes [145,146]. The modification of p21Ras likely occurs at Cys^118^ and supports a mechanism of p21Ras *S*-nitrosation, followed by an exchange reaction, which leaves p21Ras *S*-glutathionylated.

### 4.4. Myocardial Inflammation

#### 4.4.1. NFkB

The transcription factor, NFkB, a central regulator of immunity, is subject to regulation by redox changes [147]. It controls transcription of a variety of genes that regulate immune responses and inflammation [2]. *S*-glutathionylation inhibits the NFkB pathway at multiple points, including p50 [148], RelA/p65 [149] and IKK-β [51]. NFkB itself is redox-regulated, and its DNA-binding activity can be reduced by oxidative stress and activated during hypoxia [16,149]. The interaction with its inhibitor (IkB) retains NFkB in the cytoplasm in a latent inactive heterodimeric form (p65:p50 or p50:cRel) [150,151]. Once activated, NFkB translocates to the nucleus, where it binds to DNA and activates various target genes [152]. *In vivo* Glrx gene deletion sensitizes cells to oxidative inactivation of IKK-β and dampens TNFα-induced IKK and NFkB activation, resulting in varied responses to inflammation [51].

Recent evidence suggests that signaling by the proinflammatory cytokine, interleukin-1β (IL-1 β), is dependent on ROS derived from NADPH oxidase [153]. Redox signaling in response to IL-1β is known to require endocytosis of its cognate receptor (IL-1R1), followed by the formation of redox-active endosomes containing NADPH oxidase 2 (Nox2) (also called redoxosomes) [154,155]. The consequent generation of ROS by redoxosomes is responsible for the downstream recruitment of IL-1R1 effectors (IRAK, TRAF6 and IkB kinase kinases) and, ultimately, for activation of NFkB.

#### 4.4.2. Interferon Regulator Protein 3

Interferon regulatory factor 3 (IRF3) is an essential transcriptional regulator of the interferon genes. IRF3 is constitutively present in a latent conformation in the cell cytoplasm. Prinarakis *et al.* [156] reported that in human embryonic kidney (HEK) 293 cells, IRF3 is post-translationally modified by *S*-glutathionylation. Upon infection with *Sendai virus*, IRF3 undergoes deglutathionylation by the cytoplasmic enzyme, glutaredoxin-1 (GRX-1); it becomes phosphorylated, homodimerizes, translocates to the nucleus, binds to target genes and activates transcription by interacting with CBP/p300 co-activators. In virus-infected GRX-1 knockdown cells, phosphorylation, homodimerization and nuclear translocation of IRF3 do not occur; consequently, its transcriptional activity and the expression of interferon-β (IFNβ) are severely reduced. Taken together, these findings reveal a crucial role for *S*-glutathionylation in controlling the activation of IRF3 and IFNβ gene expression, with IRF3 deglutathionylation essential for transcriptional activation of the interferon genes.

## 5. Concluding Remarks

*S*-glutathionylation is a redox-dependent post-translational modification with growing relevance in signal transduction. Redox signals have been implicated in several physiological processes, including kinase signaling, channel function, apoptotic proteolysis and regulation of transcription; thus, *S*-glutathionylation may represent a “modulator” of these pathways that often cross-talk with each other. Similarly to phosphorylation, cysteine modification is critical to cellular signaling, and its deregulation has consequences over a number of human diseases, including cardiovascular diseases. Indeed, many proteins undergoing glutathionylation may contribute to the etiology of myocardial infarction, cardiac hypertrophy and atherosclerosis. In this review, we have illustrated tools and strategies to monitor protein *S*-glutathionylation and describe the proteins so far identified as glutathionylated in myocardial contraction, hypertrophy and inflammation.

## 6. Future Directions

Like the phosphorylation/dephosphorylation cycle, glutathionylation represents a mechanism by which glutathione modulates sarcomere functions depending on the tissue redox state. *S*-glutathionylation affects either phosphatases and/or kinases, thus potentially influencing phospho/dephosphorylation pathways and becoming a nexus between sulfur and phosphorous biochemistry. The cross-talk between glutathionylation and phosphorylation may be critical for the proper functioning of the cardiac proteins. The cardiac ryanodine receptor (RyR_2_), for instance, is regulated by direct phosphorylation at different sites by cAMP-dependent kinase (PKA) or calmodulin kinase II (CaMKII), and this increases the probability of the RyR_2_ channel to be open. Furthermore, SERCA_2_ is indirectly regulated by phosphorylation through the accessory protein, phospholamban (PLN), which binds to and inhibits SERCA_2_ when dephosphorylated. Even the Na^+^-K^+^ ATPase is regulated by the selective phosphorylation of phospholemman in isolated myocytes [157].

As advances in both the methodology and technology accelerate the study of glutathionylated proteins, the critical role they play in CVD is beginning to emerge. Thus, in perspective, these modifications, in combination with more standardized therapies, may offer new promise for drug development and may be used as biomarkers or predictors of cardiovascular pathology.
